# Supplementary material for: Resolving the full spectrum of human genome variation using Linked-Reads
Source: Genome Res. 2019 Apr;29(4):635–45. doi: 10.1101/gr.234443.118 (PMC6442396; doi:10.1101/gr.234443.118)
Supplement: Supplemental Material [file supp_gr.234443.118_Supplemental_longranger-2.2.zip › longranger-2.2.tar.bz2!/lr2.2-public/lib/stan/cmdstan/stan/doc/api/html/namespacemembers_func_p.html]

Stan: Namespace Members


|  |  |
| --- | --- |
|  | Stan  2.5.0  probability, sampling & optimization |


- Main Page
- Related Pages
- Namespaces
- Classes
- Files

- Namespace List
- Namespace Members

- All
- Functions
- Variables
- Typedefs
- Enumerations
- Enumerator

- a
- b
- c
- d
- e
- f
- g
- h
- i
- j
- l
- m
- n
- o
- p
- q
- r
- s
- t
- u
- v
- w


All Classes Namespaces Files Functions Variables Typedefs Enumerations Enumerator Friends Macros Pages

### - p -

- pad\_help\_option()
  : stan::io
- pareto\_ccdf\_log()
  : stan::prob
- pareto\_cdf()
  : stan::prob
- pareto\_cdf\_log()
  : stan::prob
- pareto\_log()
  : stan::prob
- pareto\_rng()
  : stan::prob
- pareto\_type\_2\_ccdf\_log()
  : stan::prob
- pareto\_type\_2\_cdf()
  : stan::prob
- pareto\_type\_2\_cdf\_log()
  : stan::prob
- pareto\_type\_2\_log()
  : stan::prob
- pareto\_type\_2\_rng()
  : stan::prob
- parse()
  : stan::gm
  , stan::json
- partial\_derivative()
  : stan::agrad
- Phi()
  : stan::agrad
  , stan::math
- Phi\_approx()
  : stan::agrad
  , stan::math
- pi()
  : stan::math
- poisson\_ccdf\_log()
  : stan::prob
- poisson\_cdf()
  : stan::prob
- poisson\_cdf\_log()
  : stan::prob
- poisson\_log()
  : stan::prob
- poisson\_log\_log()
  : stan::prob
- poisson\_rng()
  : stan::prob
- positive\_constrain()
  : stan::prob
- positive\_free()
  : stan::prob
- positive\_infinity()
  : stan::math
- positive\_ordered\_constrain()
  : stan::prob
- positive\_ordered\_free()
  : stan::prob
- pow()
  : stan::agrad
- precomputed\_gradients()
  : stan::agrad
- primitive\_value()
  : stan::agrad
  , stan::math
- print\_help\_helper()
  : stan::io
- print\_help\_option()
  : stan::io
- print\_progress()
  : stan::common
- print\_quoted\_expression()
  : stan::gm
- print\_stack()
  : stan::agrad
- print\_string\_literal()
  : stan::gm
- print\_var\_origin()
  : stan::gm
- prob\_constrain()
  : stan::prob
- prob\_free()
  : stan::prob
- prod()
  : stan::math
- promote\_common()
  : stan::math
- promote\_primitive()
  : stan::gm
- promote\_scalar()
  : stan::math


---

[ Stan Home Page ]

*© 2011–2014,
Stan Development Team.*
